# Optimizing the Delivery of mRNA to Mesenchymal Stem Cells for Tissue Engineering Applications

**DOI:** 10.1021/acs.molpharmaceut.3c00898

**Published:** 2024-03-20

**Authors:** Katie McCormick, Jorge Moreno Herrero, Heinrich Haas, Sarinj Fattah, Andreas Heise, Fergal J. O’Brien, Sally-Ann Cryan

**Affiliations:** † Tissue Engineering Research Group, Department of Anatomy and Regenerative Medicine, RCSI, Dublin D02 YN77, Ireland; ‡ School of Pharmacy and Biomolecular Sciences, RCSI, Dublin D02 YN77, Ireland; § Science Foundation Ireland Advance Materials and Bioengineering Research Centre, Dublin D02 W9K7, Ireland; ∥, BioNTech SE, Mainz 55131, Germany; ⊥ Dept. of Chemistry, RCSI, Dublin D02 YN77, Ireland; # Science Foundation Ireland Centre for Research in Medical Devices, Galway H91 W2TY, Ireland; ∇ Trinity Centre for Biomedical Engineering, Trinity College Dublin, Dublin D02 R590, Ireland

**Keywords:** mRNA, nonviral vectors, gene-activated scaffold, gene delivery, tissue engineering, mesenchymal stem cells

## Abstract

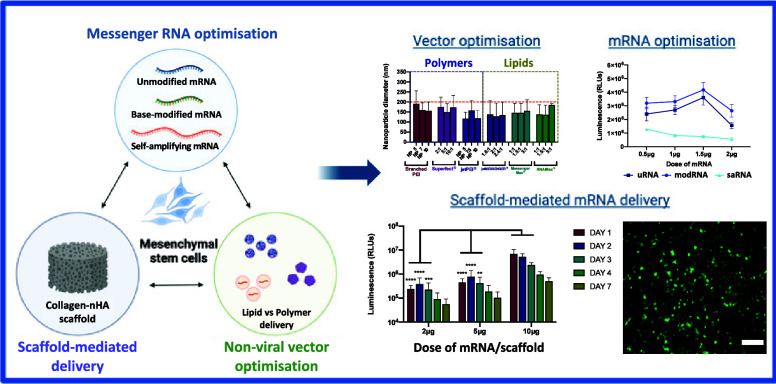

Messenger RNA (mRNA) represents a promising therapeutic tool in the field of tissue engineering for the fast and transient production of growth factors to support new tissue regeneration. However, one of the main challenges to optimizing its use is achieving efficient uptake and delivery to mesenchymal stem cells (MSCs), which have been long reported as difficult-to-transfect. The aim of this study was to systematically screen a range of nonviral vectors to identify optimal transfection conditions for mRNA delivery to MSCs. Furthermore, for the first time, we wanted to directly compare the protein expression profile from three different types of mRNA, namely, unmodified mRNA (uRNA), base-modified mRNA (modRNA), and self-amplifying mRNA (saRNA) in MSCs. A range of polymer- and lipid-based vectors were used to encapsulate mRNA and directly compared in terms of physicochemical properties as well as transfection efficiency and cytotoxicity in MSCs. We found that both lipid- and polymer-based materials were able to successfully condense and encapsulate mRNA into nanosized particles (<200 nm). The overall charge and encapsulation efficiency of the nanoparticles was dependent on the vector type as well as the vector:mRNA ratio. When screened *in vitro*, lipid-based vectors proved to be superior in terms of mRNA delivery to MSCs cultured in a 2D monolayer and from a 3D collagen-based scaffold with minimal effects on cell viability, thus opening the potential for scaffold-based mRNA delivery. Modified mRNA consistently showed the highest levels of protein expression in MSCs, demonstrating 1.2-fold and 5.6-fold increases versus uRNA and saRNA, respectively. In summary, we have fully optimized the nonviral delivery of mRNA to MSCs, determined the importance of careful selection of the mRNA type used, and highlighted the strong potential of mRNA for tissue engineering applications.

## Introduction

The field of tissue engineering and regenerative medicine aims to combine biomaterials science and biology to produce therapeutically active constructs that can mimic the native *in vivo* environment while stimulating the repair or restoration of new tissues. This generally involves the combination of the so-called “tissue engineering triad”: a scaffold construct, stem cells, and biological cues.^1^ More and more, the field is moving toward the incorporation of gene-based therapeutics to directly stimulate the differentiation, proliferation, and recruitment of stem cells within the tissue-engineered construct to further enhance the regenerative capabilities of these systems.^2−4^ In particular, mRNA (mRNA) is gaining huge interest for its gene transfer applications and is quickly beginning to supersede the use of DNA-based therapeutics within the field.^5,6^ Messenger RNA has a unique advantage in that it does not require entry into the cell nucleus in order to be functional. It is translated directly in the cell cytoplasm, which leads to faster and higher levels of protein expression versus its DNA counterpart and completely eliminates any risk of insertional mutagenesis.^7^ The recent success of mRNA-based COVID-19 vaccines has demonstrated the strong potential of mRNA-based therapeutics, and it is likely that this technology will continue to advance rapidly and pave the way for faster clinical translation into more diverse clinical applications such as tissue engineering. However, the overall stability and success of mRNA therapeutics are highly dependent on the delivery system used.

Currently, there are three main types of mRNA used in research: unmodified mRNA (uRNA), base-modified mRNA (modRNA), and self-amplifying mRNA (saRNA). Unmodified mRNA represents the most simple form of *in vitro* transcribed mRNA containing all of the essential elements of native mRNA modified in such a way to produce a stable and viable drug molecule (discussed in detail elsewhere, see refs (8 and 9)). However, one of the main drawbacks associated with uRNA is the potential to cause an immune response and activate Toll-like receptors, which overall reduces translation efficiency.^10^ In order to overcome these issues, it has been shown that the incorporation of chemically modified nucleosides such as by pseudouridine or N1-methyl-pseudouridine into the mRNA structure reduces the activation of the innate immune system.^11,12^ This type of base-modified mRNA (modRNA) is translated more efficiently and therefore has become a widely used mRNA type both in research and now clinically with two licensed COVID-19 vaccines utilizing this technology.^13,14^ The final type of mRNA is self-amplifying mRNA (saRNA), which is generally derived from alphaviruses containing the viral replicase enzyme to allow for self-amplification. This mRNA type is much larger than nonreplicating mRNA (9–12 kb) and to date has almost exclusively only been investigated for vaccine development.^15−17^ Throughout this work, we aim to screen and directly compare all three types of mRNA for their potential use in tissue engineering and determine which is best suited for our desired application.

One of the main challenges with using mRNA for tissue engineering applications is achieving effective delivery to stem cells. Mesenchymal stem cells (MSCs) are the most widely used cell type in tissue engineering due to their trilineage differentiation potential and self-renewal capabilities but have been documented as one of the most difficult to transfect cell types.^18^ In general, primary cell types are more resistant to transfection due to their finite lifespan and reduced proliferation.^19,20^ Furthermore, mRNA itself is a large (300–10,000 nt), negatively charged molecule that will not readily cross a cell membrane and can be rapidly broken down by nucleases if not adequately protected. For these reasons, nonviral gene delivery vectors are commonly employed to overcome such issues.^21,22^ Nonviral gene delivery offers advantages over viral gene delivery including an increased safety profile, reduced cost of production, and high nucleic acid loading capacities. Indeed, to date, mRNA-based therapeutics have exclusively used nonviral vectors with both EMA and FDA-approved COVID-19 mRNA vaccines utilizing lipid nanoparticles as their delivery vector of choice. Although the use of mRNA in tissue engineering is still in its infancy, a number of groups have employed nonviral vectors to achieve its delivery to stem cells. Some of the vectors that have been used for this application include branched polyethylenimine (PEI),^23,24^ lipid-based vectors,^25−27^ and lipo-polyplex materials.^28^ However, there has yet to be a comprehensive study that directly compares different vector types to determine optimal mRNA delivery conditions to MSCs. The overall aim of this study was thus to systematically screen a range of commercially available nonviral gene delivery vectors, both polymeric and lipid-based to determine which might be best suited for tissue engineering applications. Herein, we screen a variety of different vectors representing different structural architectures and chemistries used in nonviral vector development including a branched polymer (25 kDa PEI), a linear polymer (jetPEI), a dendrimer structure (Superfect), and various lipid-based materials to determine their overall suitability for mRNA delivery to stem cells. Throughout this work, we wish to determine the effect of physicochemical properties on nonviral mRNA delivery as well as determine their overall transfection efficiency, cytotoxicity, and dose required for efficient protein expression in MSCs. Furthermore, we wanted to investigate the ability of these nonviral systems to facilitate the delivery of mRNA from a collagen-based scaffold developed in our lab to support the regeneration of new tissues. In summary, this study seeks to determine optimal conditions in relation to the vector type and mRNA type for both 2D monolayer and 3D scaffold studies, as they relate to MSCs specifically. It is hoped that this work will provide insight into the interactions of mRNA with nonviral vectors and act as an optimized platform for further therapeutic mRNA applications specifically for tissue engineering.

## Materials and Methods

### Messenger RNA

In total, three different types of firefly luciferase mRNA (BioNTech SE, Germany) were used, namely, an unmodified mRNA (uRNA), a base-modified mRNA (100% N1-methyl-pseudouridine substitution), and a self-amplifying mRNA (saRNA). In addition, a dual protein-encoding (nanoluciferase and GFP) modRNA (BioNTech SE) was used in some of the later studies.

### mRNA Complex Formation

In total, six different nonviral vectors (3 polymer-based and 3 lipid-based) were screened in this study for their ability to condense and deliver mRNA to MSCs. The polymeric vectors included 25 kDa branched PEI (Sigma-Aldrich), Superfect (Qiagen), and jetPEI (Polyplus). The lipid-based vectors included jetMESSENGER (Polyplus), RNAiMAX (Invitrogen), and MessengerMax (Invitrogen). All mRNA nanoparticles were formed with each of the nonviral vectors according to the manufacturer’s instructions or according to protocols previously described by our group such as in the case of Superfect and branched PEI nanoparticles.^29,30^ As the ratio of vector:nucleic acid can have a significant impact on gene delivery efficiency, three different vector:mRNA (v/w) ratios or nitrogen/phosphate (N/P) ratios (in the case of PEI) were screened for each vector to identify optimal conditions for MSC transfection. The N/P ratio refers to the ratio of positively charged nitrogen (N) groups in a polymer-based vector to negatively charged phosphate groups (P) in the nucleic acid with which it is complexed with. Details of the ratios used as well as the complexation medium are detailed in Table 1.

**Table 1:** Complexation Conditions for mRNA-Nonviral Nanoparticles

nonviral vector	classification	vector:mRNA ratio (v/w) or N/P ratio	complexation medium	manufacturer
25 kDA branched PEI	polymer (branched)	N/P 5	nuclease-free water	Sigma-Aldrich
		N/P 7		
		N/P 10		
Superfect	polymer (activated dendrimer)	2:1	Opti-MEM	Qiagen
		5:1		
		10:1		
jetPEI	polymer (linear)	N/P 5	150 mM NaCl	Polyplus
		N/P 7.5		
		N/P 10		
jetMESSENGER	lipid	1.6:1	mRNA buffer (supplied by the manufacturer)	Polyplus
		2:1		
		2.4:1		
RNAiMax	lipid	1:1	Opti-MEM	Invitrogen
		1.5:1		
		3:1		
MessengerMax	Lipid	1:1	Opti-MEM	Invitrogen
		1.5:1		
		3:1		

### Physicochemical Characterization of mRNA Nanoparticles

#### Complex Size and Zeta Potential

For initial physicochemical characterization, 1 μg of uRNA was complexed with each of the different nonviral gene delivery vectors, Superfect, branched PEI, jetPEI, jetMESSENGER, MessengerMax, and RNAiMax, in nuclease-free water according to the manufacturer’s instructions. Samples were prepared in 50 μL volumes before being diluted to 1 mL with nuclease-free water for measurement. The complex diameter was assessed using nanoparticle tracking analysis (NTA) on a Nanosight NS 3000 instrument (Malvern, UK). NTA measurements were conducted in a static system whereby samples were loaded into a laser module sample chamber, which was maintained at 22 °C. Real-time video analysis of the nanoparticles was recorded via a built-in camera, capturing three videos per sample, with each video lasting for 60 s. For analysis, the mean size and standard deviation were calculated. For zeta potential analysis, nanoparticles were loaded into a DTS1070 Malvern folded capillary cell and analyzed using a Malvern Zetasizer ZS 3000. A total of three readings per sample, each composed of 20 submeasurements, were taken to allow for cumulative analysis. Both NTA and zeta potential measurements were independently repeated three times with freshly prepared nanoparticles.

#### mRNA Encapsulation Efficiency

The ability of each of the nonviral vectors to encapsulate mRNA was assessed using agarose gel electrophoresis. Briefly, 60 mL of a 1% agarose gel containing 6 μL of SYBR safe nucleic acid stain was prepared and submerged in a 1× Tris-borate-EDTA (TBE) buffer. Messenger RNA (uRNA) nanoparticles were prepared as described above and mixed with a 6× DNA loading dye (Thermo Scientific). Twenty μL of each sample was then loaded into wells within the gel. Controls utilized included a 1 kb DNA Plus Ladder (Life Technologies, Ireland) and uncomplexed mRNA. Electrophoresis was carried out at 80 V for approximately 45 min, and the gels were visualized using an Amersham Imager 600 (GE Healthcare, USA). Bands on the gel were quantified using ImageJ analysis.

#### Stability of mRNA Nanoparticles in the Presence of Heparin Sodium

To determine if there were any differences in the stability and dissociation potential of the mRNA nanoparticles, heparin displacement assays were performed. Nanoparticles were prepared in 50 μL volumes as described above and exposed to increasing concentrations of heparin sodium (1–10 μg) at 37 °C for 1 h. The amount of mRNA released from the nanoparticles was determined via agarose gel electrophoresis. Bands on the gel were quantified using ImageJ analysis.

### Mesenchymal Stem Cell Culture

Rat MSCs were isolated from the bone marrow of 6–8-weeks-old male Sprague–Dawley rats as previously described.^31^ Cells were cultured at a seeding density of 1 × 10^6^ cells per T175 flask until they reached 80–90% confluency. Complete MSC media consisted of Dulbecco’s modified Eagle’s medium (DMEM) supplemented with 20% fetal bovine serum (FBS), 2% penicillin/streptomycin, 1% nonessential amino acids, and 1% Glutamax (all from Sigma-Aldrich). Cells were maintained under standard cell culture conditions during all experiments (37 °C, 5% CO_2_, and 90% humidity), which were carried out using passage 5 or 6 cells.

### 2D Transfection Studies

MSCs were seeded at a density of 5 × 10^4^ cells per well in a 6-well cell culture plate 24 h prior to transfection. On the day of transfection, medium was removed and replaced with 1 mL of Opti-MEM (Gibco) for approximately 1 h while mRNA nanoparticles were being prepared as described above. The nanoparticles were then added to cells in a total volume of 500 μL of Opti-MEM. After 4 h, mRNA nanoparticles were removed, and cells were washed with 1 mL of phosphate-buffered saline (PBS) (Sigma-Aldrich) and replaced with fresh MSC growth medium.

### Reporter Protein Expression Assays

#### Firefly Luciferase

For initial 2D monolayer transfections, firefly luciferase encoding mRNAs, uRNA, modRNA, and saRNA, were used. At 24 h post-transfection, DMEM medium was removed from cells, and each well was washed with 1 mL of PBS. To detect firefly luciferase expression, a luciferase assay system (Promega, USA) was used as per the manufacturer’s guidelines. Briefly, 500 μL of a 1× reporter lysis buffer (RLB) was added to each well, and cell lysates were collected and transferred to Eppendorf tubes. For scaffold studies, each scaffold was submerged in 500 μL of 1× RLB in an Eppendorf tube. To achieve complete cell lysis, the cells/scaffolds were subjected to one freeze–thaw cycle prior to analysis. Twenty μL of each lysate was then added to a white 96-well plate and mixed with 100 μL of a luciferase assay reagent via pipetting. The plate was transferred immediately to a plate reader, and luminescence was recorded for each well in triplicate.

#### Nanoluciferase and GFP

Following the identification of optimal vectors and mRNA types, a modRNA encoding both nanoluciferase (nLuc) and GFP was used. Nanoluciferase is a secreted protein and can be detected in cell culture media. Media samples were collected at various time points and analyzed for nLuc activity using a Nano-Glo luciferase assay system (Promega) according to the manufacturer’s instructions. Briefly, 10 μL of each media sample was plated in triplicate into a white 96-well plate and diluted to a final volume of 100 μL with deionized water. A Nano-Glo luciferase assay reagent was prepared by diluting the Nano-Glo luciferase assay substrate with 50 volumes of the assay buffer supplied by the manufacturer. One hundred μL of the reagent was then added to each sample in the 96-well plate and incubated at room temperature for 3 min before analyzing luminescence on a Tecan Infinite 200 Pro plate reader. GFP expression was also analyzed in the same samples at 24 h using a fluorescence Leica microscope.

### Biocompatibility of mRNA Nanoparticles in a 2D Monolayer

To determine the viability of cells post-transfection with mRNA particles, MTS assays were conducted using a CellTiter 96 Aqueous Assay (Promega). For this assay, rMSCs were seeded at a density of 1 × 10^4^ cells per well in a 96-well cell culture plate. Messenger RNA nanoparticles were prepared as previously described using a lower dose of 0.2 μg of modRNA. The nucleic acid dose used reflects a dose adjustment from 5 × 10^5^ cells/well on a 6-well plate assay to 1 × 10^4^ cells/well in a 96-well plate. Following a 4 h transfection process, as described above, each plate was washed and incubated in the presence of complete MSC medium for 24 and 72 h. At each time point, 20 μL of the MTS assay reagent was added to each well, and the plate was incubated for a further 2 h at 37 °C. The absorbance of each well was determined at 490 nm. The final cell viability is expressed as a percentage of the untreated control group (100% cell viability).

### Optimization of Scaffold-Based mRNA Delivery

#### Collagen-Nanohydroxyapatite (nHA) Scaffold Fabrication

Collagen-nanohydroxyapatite scaffolds used throughout this work were fabricated according to a previously optimized technique developed by our group.^32,33^ A 0.5% w/v collagen slurry was formed via the addition of type 1 bovine collagen (SLB, New Zealand) to 0.05 M glacial acetic acid with blending for 90 min. An nHA suspension was created using a novel dispersant-aided precipitation technique as previously described.^34^ The resulting nHA suspension was centrifuged, and the supernatant was removed before being resuspended in 30 mL of distilled water with sonication. The nHA suspension was then added slowly to the blending collagen slurry, and blending was continued for a further 3 h to form a 100 wt % nHA slurry. Following slurry formation, gas was removed using a vacuum pump, and 400 μL was pipetted into individual scaffold 10 mm stainless-steel molds and freeze-dried to a final temperature of −40 °C. Post lyophilization, the newly formed scaffolds were cross-linked dehydrothermally (DHT) at 105 °C for 24 h at 0.05 bar in a vacuum oven (Vacucell 22; MMM, Germany), which functions to both sterilize and structurally reinforce the scaffolds. Prior to use, scaffolds were rehydrated in descending grades of ethanol followed by PBS. The scaffolds were then chemically cross-linked using a mixture of *N*-(3-(dimethylamino)propyl)-*N*′-ethylcarbodimide hydrochloride (EDAC) and *N*-hydroxysuccinimide (NHS) (Sigma-Aldrich) in a ratio of 5:2 for 2 h at room temperature.^35^ Scaffolds were rinsed three times in PBS before use on the cells.

#### mRNA-Activated Scaffold Formation and 3D Transfection

To form an mRNA-activated scaffold, mRNA nanoparticles were formed as described above in 50 μL volumes. Each scaffold was placed in a well of a 24-well suspension tissue culture plate, and 25 μL of mRNA nanoparticle solution was soak-loaded onto one side of the scaffold. The scaffolds were then incubated at 37 °C for 15 min. Following incubation, 2 × 10^5^ rMSCs, cultured as previously described, were added to the same side of the scaffold in 25 μL of Opti-MEM. The scaffolds were incubated for a further 15 min at 37 °C. Following this incubation, the scaffolds were carefully flipped using sterile forceps, and the above steps were repeated on the other side of each scaffold. This soak-loading procedure was based on protocols previously described by our lab using other nucleic acid cargos.^4,36,37^ By the end of the process, each scaffold had been soak-loaded with 50 μL of mRNA nanoparticles and 50 μL of the cell suspension. One mL of Opti-MEM was then added to each well containing a scaffold, and the plate was incubated under standard culture conditions for 24 h. After 24 h, the scaffolds were transferred to a new 24-well suspension plate, and the Opti-MEM was replaced with standard rMSC media. The plate was then incubated under standard cell culture conditions, and samples were taken at designated time points.

#### Biocompatibility of mRNA-Activated Scaffolds

The cytocompatibility of the mRNA-activated scaffolds was assessed using an Invitrogen LDH cytotoxicity assay (Fisher Scientific) and Invitrogen Quanti-iT PicoGreen assays (Fisher Scientific). The LDH assays were carried out on supernatants collected at days 1, 3, and 7 post-transfection. Briefly, 50 μL of each sample was pipetted into a clear 96-well plate in triplicate and mixed with 50 μL of an LDH reaction mixture, which was freshly prepared according to the manufacturer’s instructions. The plate was incubated for 30 min at room temperature, and absorbance was read at 490 and 680 nm using a Tecan Infinite 200 Pro plate reader. The LDH activity was determined by subtracting the 680 nm absorbance values from the 490 nm absorbance values and expressed as a percentage versus untreated rMSCs.

For PicoGreen assays, scaffolds were removed from media and washed briefly in PBS. The scaffolds were then submerged in 500 μL of a lysis buffer (0.2 M carbonate and 1% Triton X-100) in an Eppendorf. Samples were subjected to three freeze–thaw cycles to achieve complete cell lysis prior to analysis. One hundred μL of each sample was then transferred to a black 96-well plate along with 100 μL of a PicoGreen reagent. Fluorescence was read at 538 nm, and the final DNA concentration was calculated from a standard curve generated using standards formulated according to the manufacturer’s instructions.

#### Confocal Imaging of mRNA-Activated Scaffolds

To assess the distribution of mRNA nanoparticles within a collagen-nHA scaffold, modRNA was tagged with Cy3 using a Mirus Label IT Cy3 nucleic acid labeling kit (Medical Supply Company, Ireland) according to the manufacturer’s instructions. The tagged modRNA (5 μg) was complexed with jetPEI (2:1 v/w) and soak-loaded onto both sides of the collagen-nHA scaffolds. Scaffolds were imaged on a Carl Zeiss LSM 710, equipped with a W Plan-Apochromat 20× (NA 1.0) with an interslice *Z* spacing of 1.2 μm to yield a total image *Z* depth of 31.2 μm. The scaffold autofluorescence was excited using a 405 nm laser (detection range of 410–509 nm). Cy3 fluorescence was excited using a 561 nm laser (detection range of 564–681 nm). Images were recorded at a resolution of 1024 × 1024 pixels with a dwell time of 0.79 μs. Z stack images were maximum intensity projected and prepared in ImageJ. To assess the initial depth of particle incorporation within the scaffold, the scaffolds were also sectioned with a scalpel to expose the center of the scaffold, which was then imaged as described above. ImageJ software was used to determine the depth of the nanoparticle infiltration into the scaffold.

#### Immunogenicity of mRNA-Activated Scaffolds

To determine the potential of mRNA nanoparticles to exhibit an immunogenic response, mRNA nanoparticles were loaded onto collagen-nHA scaffolds as described above along with 1 × 10^6^ human peripheral blood mononuclear cells. Human peripheral blood mononuclear cells (hPBMCs) were isolated from fresh human blood (*n* = 3 donors) and resuspended in RPMI-GlutaMax media containing 10% FBS and 1% penicillin/streptomycin (Sigma-Aldrich). The hPBMCs were loaded onto mRNA-activated scaffolds (1 × 10^6^ cells/scaffold) and incubated under standard cell culture conditions for 24 h. At this time point, cell supernatants were collected and analyzed for IL-6, IL-8, and TNF-α contents using ELISAs according to the manufacturer’s instructions. (IL-6 and IL-8 ELISAs, Biolegend; TNF-α ELISA, R&D Systems).

### Statistical Analysis

Results in this study are expressed as means ± standard deviation of three independent repeats. Statistical analysis was performed using GraphPad Prism version 9.0 (GraphPad Software, CA, USA). One-way ANOVA tests were performed to compare differences between multiple groups followed by Tukey’s *post hoc* analysis unless otherwise stated. Significance was determined using *P* values of * < 0.05, ** < 0.01, *** < 0.001, and **** < 0.0001.

## Results

### Physicochemical Analysis of mRNA Nanoparticles

#### Zeta Potential and Size of mRNA Nanoparticles

**Figure 1:**
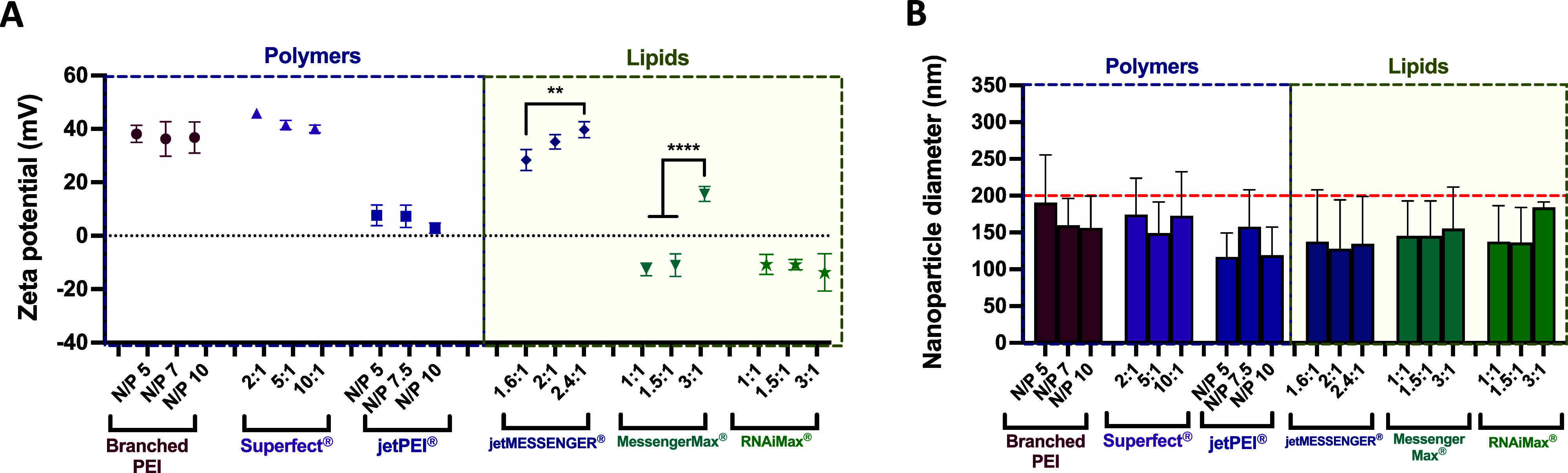
Physicochemical analysis of mRNA nanoparticles with nonviral vectors. One μg of unmodified mRNA (uRNA) was complexed with both polymeric and lipid-based vectors across a range of vector:mRNA (v/w) or nitrogen/phosphate (N/P) ratios according to the manufacturer’s instructions. The nanoparticles were characterized in terms of zeta potential (mV) (A) and size (nm) (B). Results are expressed as means ± standard deviation (*n* = 3) where **p < 0.01 and ****p < 0.0001. The red hatched line in (B) indicates a size of 200 nm.

Each of the nonviral vectors was complexed with uRNA at different ratios and analyzed in terms of size and zeta potential (Figure 1). A range of vector:mRNA ratios were initially screened as this has long been established as a determining factor for nucleic acid complexation and ultimately transfection efficiency in nonviral gene delivery.^38,39^ The range of N/P or mass ratios chosen for each vector was guided by the manufacturer’s recommendations or previously published work on the vector systems.^29,30^ For polymeric materials (branched PEI, Superfect, and jetPEI), all mRNA nanoparticles formed were positively charged, indicating complete complexation of the nucleic acid within the cationic materials (Figure 1A). Superfect-mRNA nanoparticles showed the highest overall charge with a range of 40–45.9 mV, whereas jetPEI-mRNA nanoparticles were much less cationic with a range of 2.9–7.7 mV. The lipid-based materials showed more varied results in terms of the zeta potential with jetMESSENGER-mRNA nanoparticles displaying a high cationic charge (28.4–39.7 mV), and the mRNA nanoparticles formed with the other two Lipofectamine reagents (MessengerMax and RNAiMax) having an overall negative charge with the exception of a 3:1 v/w ratio for MessengerMax. In terms of size, all nanoparticles, regardless of the vector or ratio used to complex the mRNA, had a size less than 200 nm (Figure 1B) in diameter as determined by nanoparticle tracking analysis (NTA). This nanoparticle size has been previously quoted as favorable for facilitating MSC uptake and transfection.^40^ The polydispersity index (PDI), which provides additional information about the size distribution of the mRNA nanoparticles, is provided in the Supporting Information (Table S1).

#### Encapsulation Efficiency and Stability of mRNA Nanoparticles

**Figure 2:**
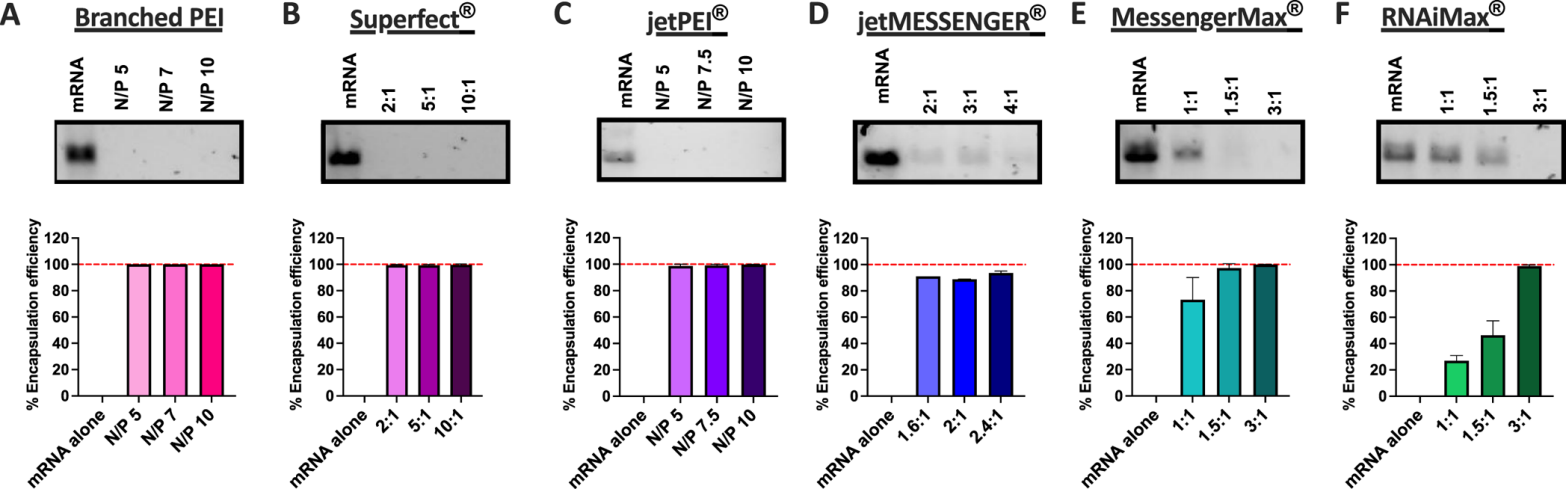
Encapsulation efficiency of various nonviral vectors with mRNA. A range of nonviral vectors were complexed with 1 μg of uRNA across three different vector:mRNA (v/w) or nitrogen/phosphate (N/P) ratios and assessed for encapsulation efficiency using agarose gel electrophoresis. Encapsulation efficiency versus uncomplexed mRNA (%) was determined by using ImageJ software. All polymeric nanoparticles ((A) branched PEI, (B) Superfect, and (C) jetPEI) achieved full mRNA encapsulation, as indicated by clear lanes on the gels. Lipid-based vectors ((D) jetMESSENGER, (E) MessengerMax, and (F) RNAiMax) showed more varied encapsulation with a general increase in encapsulation with an increasing vector:mRNA (v/w) ratio. Hatched red lines indicate 100% encapsulation.

The ability of the vector systems to condense and encapsulate mRNA was determined by using agarose gel electrophoresis (Figure 2). All polymeric systems fully encapsulated the mRNA across a range of N/P or mass ratios (v/w), which was indicated by the absence of bands on the agarose gels (Figure 2A–C). This was to be expected given the cationic charge of the nanoparticles formed in Figure 1A. The lipid-based vectors showed varying degrees of encapsulation, with a trend for increasing encapsulation with an increase in vector concentration. For example, the RNAiMax vector only had a 29 ± 3% encapsulation efficiency at a 1:1 v/w ratio but was able to achieve 100% encapsulation at a 3:1 v/w ratio, indicating that the vector:mRNA ratio has an important impact on mRNA condensation. Due to the low encapsulation of mRNA with both Lipofectamine reagents (MessengerMax and RNAiMax) at the 1:1 v/w ratio, this ratio was excluded from further screening studies.

**Figure 3:**
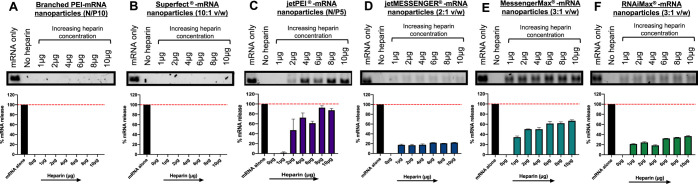
Dissociation potential of nonviral mRNA nanoparticles in the presence of heparin sodium. Nonviral vectors were complexed with 1 μg of uRNA ((A) branched PEI-mRNA N/P 10, (B) Superfect-mRNA 10:1, (C) jetPEI-mRNA N/P 5, (D) jetMESSENGER-mRNA 2:1 v/w, (E) MessengerMax-mRNA 3:1 v/w, and (F) RNAiMax-mRNA 3:1 v/w) and exposed to increasing concentrations of heparin sodium (1–10 μg) for 1 h at 37 °C. Subsequent mRNA release was determined using agarose gel electrophoresis followed by quantification of band intensity using ImageJ analysis. Red hatched lines indicate 100% mRNA release.

To determine if there were any differences in the stability and dissociation of the nanoparticles, heparin displacement assays were performed (Figure 3). Heparin is a large, anionic polysaccharide that has the ability to compete with nucleic acid binding and disrupt complex stability.^41^ Previous studies have indicated that the binding strength and release of a nucleic acid from its vector can influence the overall transfection efficiency.^42,43^ In this study, mRNA nanoparticles were prepared and incubated with increasing concentrations of heparin sodium at 37 °C for 1 h. The N/P or mass ratio used for each vector system is detailed in Figure 3. The amount of mRNA released was then analyzed using agarose gel electrophoresis. There was no evidence of mRNA release from either the branched PEI or Superfect-mRNA nanoparticles (Figure 3A,B) even at high concentrations of heparin sodium indicating a higher binding strength to the mRNA versus other vector systems. jetPEI was the only polymeric vector that showed significant release of the mRNA in the presence of heparin sodium achieving up to 93% release at higher doses (8–10 μg) of heparin. All lipid-based vectors demonstrated some level of mRNA release in the presence of heparin, which varied depending on the vector. MessengerMax-mRNA nanoparticles showed the highest mRNA release among the lipid vectors with ∼67% of the cargo released at a 10 μg heparin dose, whereas ∼22% mRNA release was observed from jetMESSENGER-mRNA nanoparticles at the same heparin dose.

### Optimization of Mesenchymal Stem Cell Transfection with mRNA Nanoparticles

**Figure 4:**
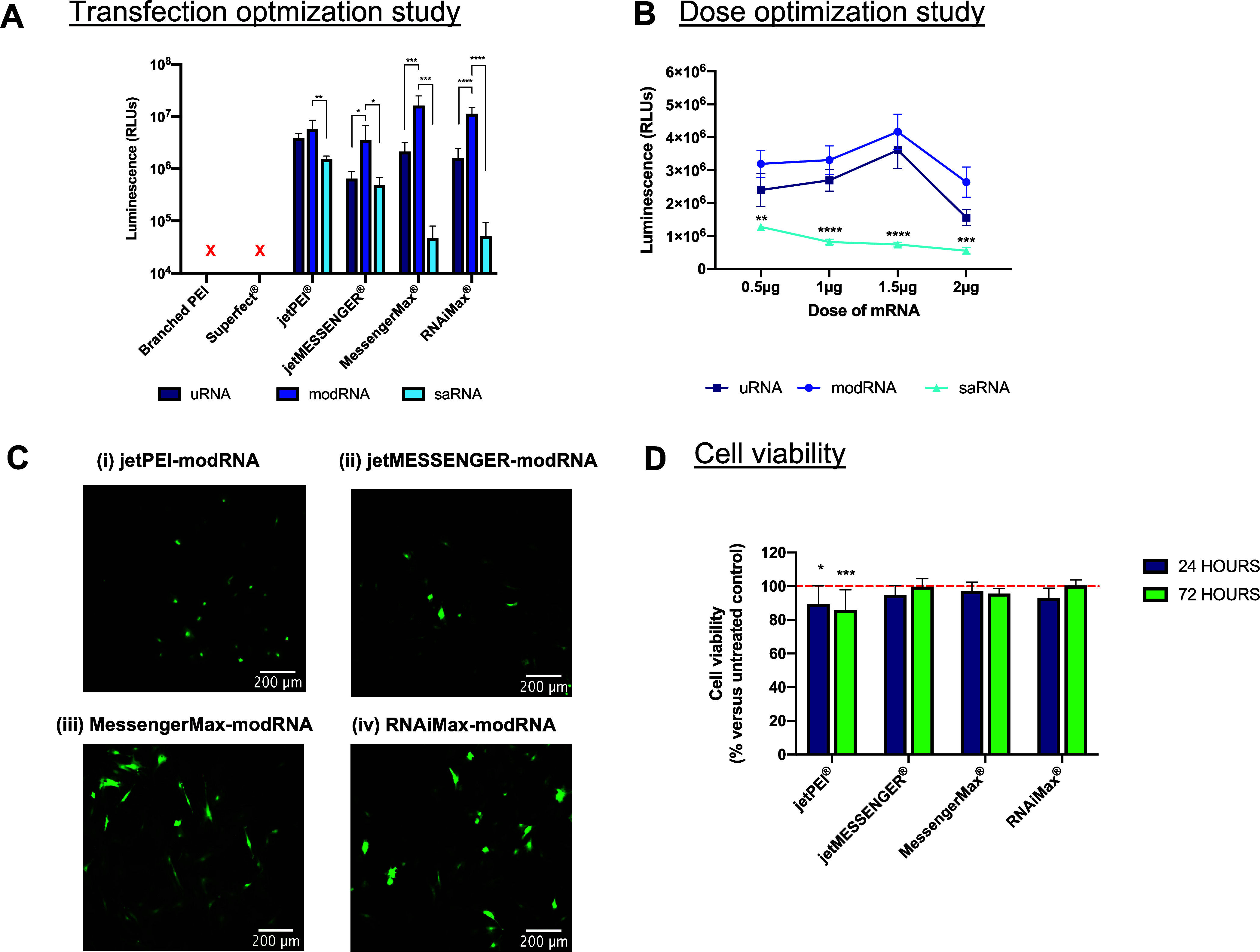
Investigating the effect of the mRNA type and vector type for MSC transfection in 2D monolayer culture. (A) Three different types of mRNA (1 μg); unmodified (uRNA), base-modified (modRNA), and self-amplifying (saRNA) were complexed with a range of nonviral vectors and assessed for luciferase expression at 24 h post-transfection. Vector:mRNA ratios used included N/P 5 for jetPEI, 2:1 v/w for jetMESSENGER-mRNA nanoparticles, and 3:1 v/w for MessengerMax and RNAiMax-mRNA nanoparticles. (B) Four different doses of mRNA (0.5, 1, 1.5, and 2 μg) were investigated to determine the optimal dosage for each mRNA type. (C) modRNA (1 μg) encoding GFP was delivered using the four lead vectors and analyzed at 24 h using fluorescence microscopy. (D) Cell viability following transfection with optimized modRNA (1 μg) nanoparticles was assessed at 24 and 72 h using the MTS assay. The red hatched line indicates 100% cell viability (untreated cells). Where relevant, results are expressed as means ± SD (*n* = 3) where *p < 0.05, **p < 0.01, ***p < 0.001, and ****p < 0.0001. For (B), significance is shown at each dose in comparison to modRNA nanoparticles. For (D), significance is shown in comparison to untreated cells at each time point.

To gain a more comprehensive understanding of the impact of the vector type and mRNA type on MSC delivery, a direct comparison study was conducted (Figure 4). All six vectors (branched PEI, Superfect, jetPEI, jetMESSENGER, MessengerMax, and RNAiMAX) were complexed with 1 μg of each of the three mRNA types (uRNA, modRNA, and saRNA) at their optimized ratio (see Supporting Information, Figure S1) and directly compared in terms of luciferase expression (reported as RLUs) 24 h post-transfection (Figure 4A). Neither of the two branched polymeric materials, branched PEI and Superfect, showed any significant levels of luciferase expression with any of the three mRNA types. jetPEI was the only polymer-based material that resulted in significant luciferase expression. When the three mRNA types were compared, overall, modRNA demonstrated significantly higher luciferase expression versus both uRNA and saRNA regardless of the vector used. In terms of determining the optimal vector for modRNA delivery, MessengerMax-modRNA nanoparticles achieved the highest luciferase levels overall (1.6 × 10^7^ ± 8.6 × 10^6^ RLUs), which was significantly higher versus the jetPEI-modRNA (*p* < 0.01) and jetMESSENGER-modRNA groups (*p* < 0.001). Overall, there was no significant difference between MessengerMax and RNAiMax (1.1 × 10^7^ ± 3.8 × 10^6^ RLUs) when used to deliver modRNA to MSCs. In contrast, for both uRNA and saRNA delivery, jetPEI was the most successful vector, achieving significantly higher transgene expression versus all other vector systems for both of these mRNA types.

To evaluate the effect of the mRNA dose on protein expression, jetPEI-mRNA (N/P 5) nanoparticles were formed across four different doses (0.5, 1, 1.5, and 2 μg) with the three mRNA types (Figure 4B). jetPEI was chosen for this study, as it provided the most consistent results for all three mRNA types (as per Figure 4A). Both uRNA and modRNA demonstrated remarkably similar dose–response patterns. There was a consistent increase in protein expression for both mRNA types with an increasing mRNA dose, reaching a peak at 1.5 μg. Beyond this dose, protein expression significantly decreased for both mRNA types at a 2 μg dose (*p* < 0.05 modRNA and *p* < 0.001 uRNA), suggesting a potential saturation effect. In contrast, saRNA nanoparticles demonstrated significantly lower protein expression profiles versus uRNA and modRNA with luciferase expression peaking at 0.5 μg and showing a general decline with an increasing dose. Overall, modRNA demonstrated the highest protein expression levels across the range of doses achieving up to 1.2-fold and 5.6-fold increases in luciferase expression (at the 1.5 μg dose) versus uRNA and saRNA, respectively. From these results, we could conclude that the modRNA nanoparticles are optimal for MSC transfection in terms of achieving the highest levels of protein expression.

To further validate these results, the four lead vectors (jetPEI, jetMESSENGER, MessengerMax, and RNAiMax,) were then used to deliver a GFP encoding modRNA at their optimized vector:mRNA ratios (N/P 5 for jetPEI, 2:1 v/w for jetMESSENGER, and 3:1 v/w for MessengerMax and RNAiMax) (Figure 4C). The GFP expression assays indicated that the mRNA nanoparticles formed using lipid-based vectors, in particular MessengerMax and RNAiMax, transfected cells more efficiently than jetPEI and jetMESSENGER as evidenced by a greater number of green fluorescent cells (Figure 4C). The cell viability of the lead modRNA nanoparticles was then assessed at 24 and 72 h post-transfection (Figure 4D). All mRNA nanoparticles achieved at least 85% cell viability versus untreated control cells at both time points. Furthermore, there was no significant decrease in viability between 24 and 72 h with any vector-mRNA nanoparticle indicating no prolonged toxicity associated with any of the mRNA nanoparticles.

### Investigating the Ability of a Collagen-Nanohydroxyapatite Scaffold to Deliver mRNA Nanoparticles

**Figure 5:**
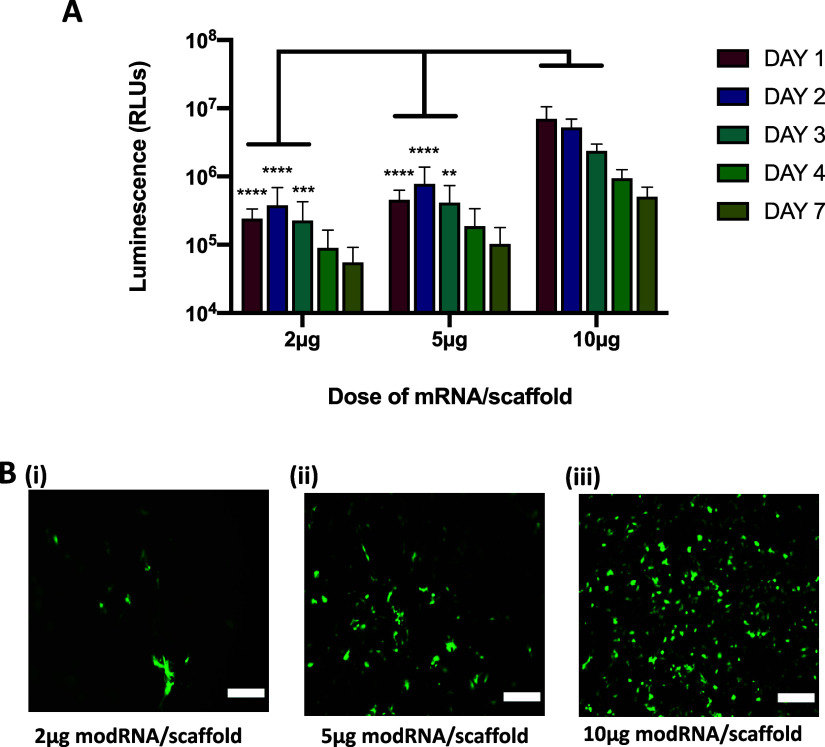
mRNA-activated scaffold transgene expression profile. jetPEI-modRNA nanoparticles (N/P 5) encoding nanoluciferase and GFP protein were loaded onto our collagen-nHA scaffold across a range of doses (2, 5, and 10 μg). (A) Secreted nanoluciferase was analyzed in media samples collected over 7 days post-treatment. (B) GFP expression within the scaffold was assessed using fluorescence microscopy 24 h post-treatment. Where relevant, results are expressed as means ± SD (*n* = 3) where **p < 0.01, ***p < 0.001, and ****p < 0.0001. Scale bar = 200 nm.

The field of tissue engineering relies heavily on the use of biomaterial scaffolds to act as templates for new tissue growth while simultaneously acting as a depot for the delivery of biological cues such as recombinant proteins or, in this case, gene-based therapeutics. When developing a gene-activated scaffold system for tissue repair, the scaffold system must be able to support the loading and efficient delivery of particles to the surrounding cells. In order to interrogate this for mRNA delivery, we chose a collagen-nanohydroxyapatite scaffold (nHA) that has been optimized and has shown proven potential for bone repair as a proof of concept for this study.^34,36^ Collagen-nHA scaffolds were soak-loaded on both sides with jetPEI-modRNA nanoparticles encoding GFP and nanoluciferase across a range of doses (2, 5, and 10 μg). These gene-activated scaffolds were then seeded with 4 × 10^5^ MSCs (2 × 10^5^ MSCs/side of scaffold). Secreted luciferase levels were analyzed over the course of 7 days (Figure 5A), and GFP expression was determined using fluorescent microscopy at 24 h post soak-loading (Figure 5B). Overall, we see an increasing amount of protein expression with increasing doses of mRNA when loaded onto the collagen-nHA scaffold. For example at 24 h, the 10 μg mRNA-loaded scaffolds produced significantly higher expression (6.2 × 10^7^ RLUs) versus the 2 μg mRNA scaffold group (1.4 × 10^6^ RLUs) (*p* < 0.0001). These results can also be seen in the GFP images taken at 24 h with the 10 μg mRNA-loaded scaffolds showing a higher amount of green fluorescent cells versus the other two groups using lower mRNA doses (Figure 5B). Over the course of 7 days, the amount of protein expressed decreases gradually in each group, which is to be expected with mRNA due to the fast translation of this gene therapeutic intracellularly. In summary, however, these results are encouraging and indicate that the scaffolds can support the transfection of MSCs with modRNA nanoparticles and act as a depot for the release of secreted protein over the course of at least 7 days.

### Distribution of mRNA Nanoparticles within a Collagen-nHA Scaffold

**Figure 6:**
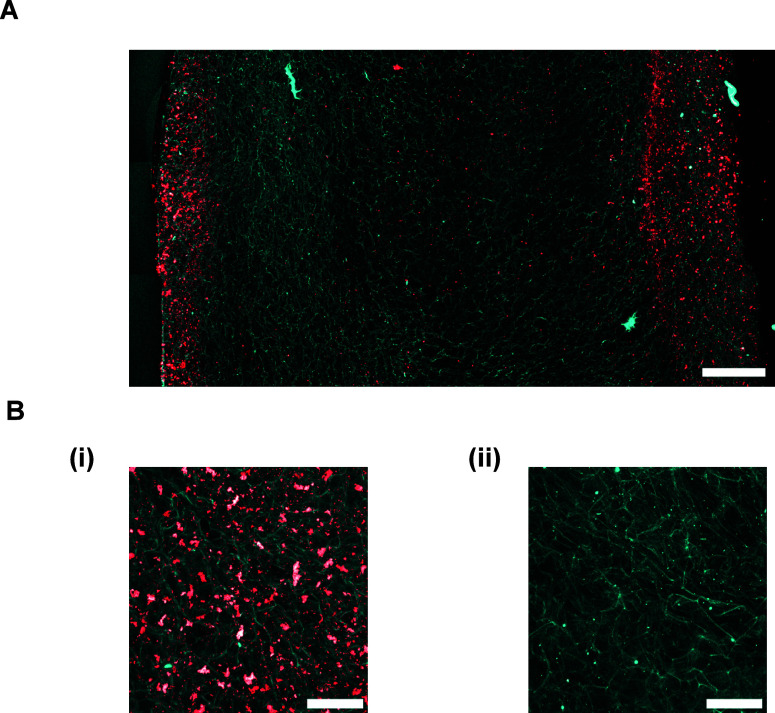
Distribution of mRNA nanoparticles in a collagen-nHA scaffold. (A) Representative confocal scanning micrograph of Cy3-labeled mRNA nanoparticles (jetPEI-modRNA 5 μg 2:1 v/w) in a cross section of a collagen-nHA scaffold. mRNA nanoparticles are fluorescently tagged (red), and the collagen-nHA scaffold structure autofluoresces (blue). mRNA nanoparticles remained close to both scaffold surfaces, where they had initially been soak-loaded. Scale bar = 500 μm. (B) Higher-magnification confocal scanning micrograph of (i) Cy3-labeled mRNA nanoparticles within the nanoparticle-rich area of the scaffold demonstrating even distribution of nanoparticles throughout the scaffold pores. (ii) A blank collagen-nHA scaffold was imaged as a control. Scale bar = 100 μm.

To investigate the distribution of mRNA nanoparticles within a scaffold structure, Cy3-labeled mRNA nanoparticles (jetPEI-modRNA 5 μg 2:1 v/w) were soak-loaded onto both sides of a collagen-nHA scaffold and imaged using confocal microscopy (Figure 6). The scaffolds were bisected using a scalpel to allow visualization of a full cross section of the structure and determine the depth of nanoparticle infiltration (Figure 6A). Fluorescently tagged mRNA nanoparticles (red) can be clearly seen evenly distributed across both surfaces of the collagen-nHA scaffold that autofluoresces (blue). From the images, it appears that the majority of mRNA nanoparticles remain close to the surface of the scaffold structure where they were initially soak-loaded with very few nanoparticles detected toward the center of the scaffold. Using ImageJ software, it was estimated that the nanoparticles infiltrated approximately 0.3–0.8 mm of the scaffold’s total depth (∼4.6 mm) on each side.

Higher-magnification images were also taken of the Cy3 mRNA-loaded scaffold closer to the scaffold surface, where there was a high density of tagged mRNA nanoparticles (Figure 6B(i)). From the images, a uniform and widespread distribution of nanoparticles (red) can be seen throughout the scaffold’s pore structure (blue) in these nanoparticle-dense areas. A blank collagen-nHA scaffold served as a control in this study, which only showed the autofluorescence (blue) of the scaffold itself (Figure 6B(ii)).

### Optimization of the mRNA:Vector Formulation for Transfection of MSCs in Collagen-nHA Scaffolds

**Figure 7:**
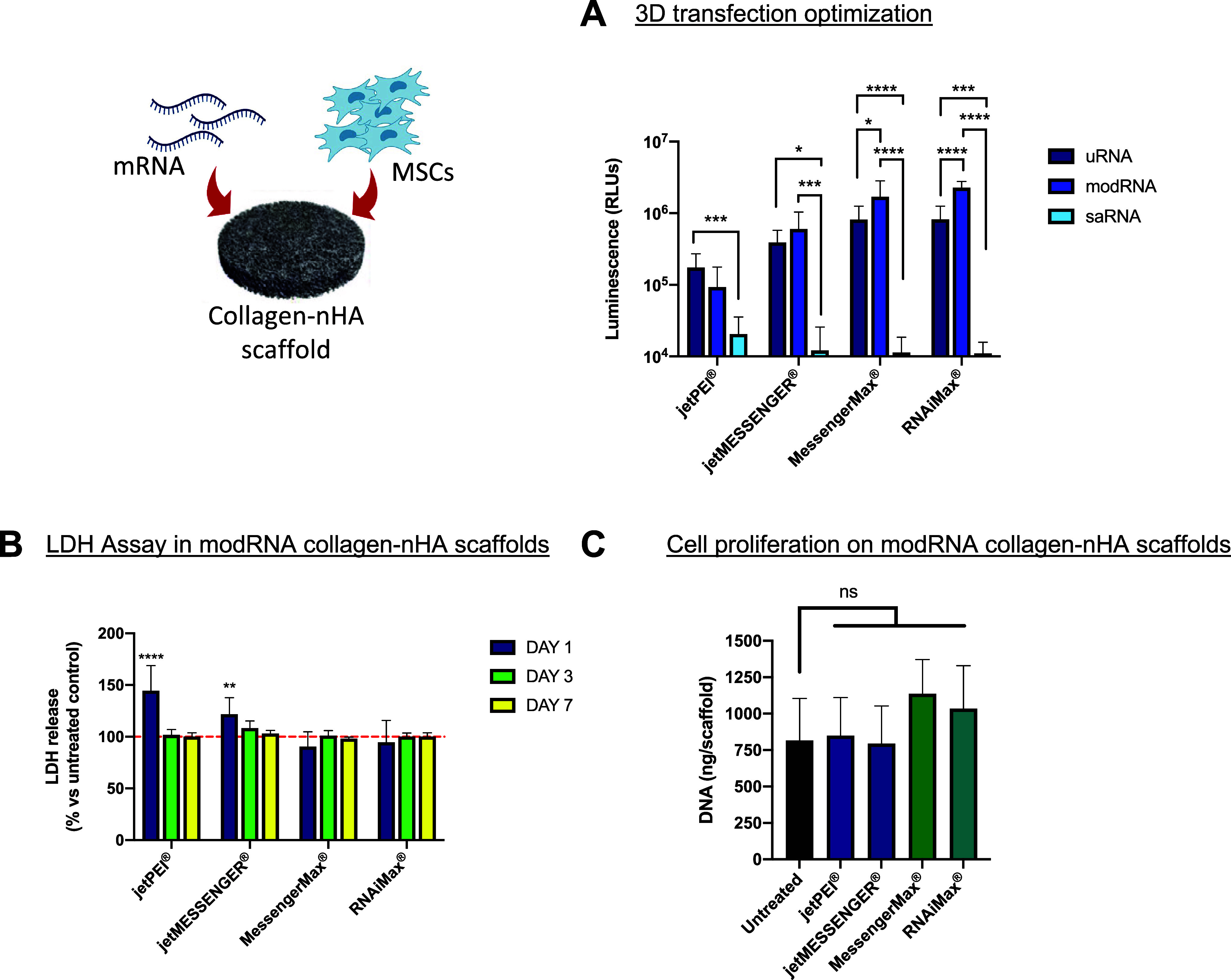
Optimizing mRNA transfection of MSCs from a collagen-nanohydroxyapatite scaffold. Two μg of each mRNA type (uRNA, modRNA, and saRNA) was complexed with the four lead vectors (jetPEI N/P 5, jetMESSENGER 2:1 v/w, MessengerMax 3:1 v/w, and RNAiMAX 3:1 v/w) and soak-loaded onto a collagen-nanohydroxyapatite (nHA) scaffold. Media were collected at 24 h and analyzed for the luciferase content (A). To assess cell viability of modRNA (2 μg) scaffolds, LDH release was analyzed at days 1, 3, and 7 (B), and cell proliferation was determined 7 days post-treatment using DNA quantification (C). The red hatched line in (B) indicates 100% cell viability (untreated cells). Results are expressed as means ± SD (*n* = 3) where *p < 0.05, **p < 0.01, ***p < 0.001, and ****p < 0.0001. Statistics shown in (B) and (C) represent statistical significance versus untreated cells.

Having confirmed the ability to transfect MSCs effectively using nanoparticles loaded onto a collagen-nHA scaffold, we next confirmed an optimal vector system for this application. Previous studies of nucleic acid scaffold systems have indicated that protein expression achieved in 2D can vary greatly when translated to a 3D scaffold system.^29,44^ Because of this, our four lead vectors (jetPEI, jetMESSENGER, RNAiMax, and MessengerMax) were once again screened with the three mRNA types in order to determine optimal conditions for mRNA delivery from the scaffold system (3D) (Figure 7A). Similar to what was observed in 2D studies, the lipid-based materials, in particular RNAiMax-modRNA nanoparticles and MessengerMax-modRNA nanoparticles, had the highest levels of protein expression overall in MSCs seeded onto the collagen-nHA scaffolds (∼2.3 × 10^6^ and ∼1.7 × 10^6^ RLUs, respectively). Both RNAiMax-modRNA and MessengerMax-modRNA nanoparticles achieved significantly higher (*p* < 0.0001) levels of reporter protein expression versus jetPEI-modRNA (∼9.3 × 10^4^ RLUs) and jetMESSENGER-modRNA (∼6 × 10^5^ RLUs) nanoparticles when delivered on a collagen-nHA scaffold.

Modified mRNA resulted in the highest luciferase expression in all groups, with the exception of jetPEI. In the jetPEI groups, the jePEI-uRNA nanoparticles led to the highest transfection levels. Overall, the saRNA resulted in significantly lower luciferase expression in all groups, indicating that it may not be ideally suited for MSC transfection.

The effect of the nanoparticle composition on the MSC cell viability within the collagen-nHA scaffold was then assessed. Each vector was complexed with modRNA and soak-loaded with MSCs onto the scaffold. The LDH content was then analyzed at 24 and 72 h post soak-loading (Figure 7B). LDH is a cytosolic enzyme that is released into the cell culture medium upon damage to the cell membrane. Therefore, a higher LDH content can be an indicator of cellular toxicity. Both jetPEI-modRNA and jetMESSENGER-modRNA nanoparticles showed significantly higher LDH production versus untreated cells at 24 h, while the two lead vector formulations (MessengerMax-modRNA and RNAiMax-modRNA) showed no significant increase in LDH. Cell proliferation within the scaffolds was also assessed by measuring the DNA content 7 days post soak-loading (Figure 7C). None of the four vector-mRNA nanoparticles showed a decrease in the DNA content within the scaffold with the mRNA nanoparticles composed of the two Lipofectamine reagents (MessengerMax and RNAiMax) showing a slight increase in cell proliferation versus an untreated scaffold, although this was not significant.

Finally, a preliminary study to investigate any potential immunogenic response of an mRNA-activated scaffold was conducted using human peripheral blood mononuclear cells (hPBMCs) 24 h postexposure to the modRNA-activated scaffolds (see Supporting Information, Figure S2). For this study, one lead polymer-based vector (jetPEI) and one lead lipid-based vector (MessengerMax) were chosen and complexed with modRNA across two different doses (2 and 5 μg). Overall, jetPEI-modRNA-loaded scaffolds showed a general trend of increased IL-6, IL-8, and TNF-α at 24 h versus MessengerMax-modRNA-loaded scaffolds although this was only statistically significant in the TNF-α 2 μg group (*p* < 0.05). Interestingly, for both vector formulations, increasing the dose of modRNA did not appear to significantly increase the amount of cytokines produced.

Taken together, we can conclude that lipid-based vectors are the most efficient for the delivery of mRNA from our collagen-nHA scaffolds with no significant effects on cell viability or proliferation of MSCs, and modRNA nanoparticles achieve the highest protein expression levels overall.

## Discussion

Messenger RNA represents a promising gene transfer option for the efficient and fast delivery of growth factors to support the regeneration of new tissues. The platform can be used for the delivery of single or multiple genes depending on the desired application.^5,25,45^ Therefore, the development of protocols for optimized mRNA transfection is of utmost importance in order to further progress basic as well as applied translational research in this area. Throughout this work, we describe the optimization of mRNA delivery to MSCs for tissue engineering applications both in a 2D monolayer and from a 3D collagen-based scaffold. We screened a range of commercially available lipid and polymeric vector systems, as well as multiple mRNA types to determine the impact of both factors on MSC transfection efficiency. We chose a variety of vectors representing the diverse molecular architectures that are currently used in nonviral gene delivery including a branched polymer (25 kDa PEI), a linear polymer (jetPEI), a dendrimer (Superfect), and a range of lipid-based vectors that have been specifically designed for RNA delivery (jetMESSENGER, MessengerMax, and RNAiMax). Overall, we found that lipid-based materials combined with modified mRNA provide an optimal platform for MSC transfection in terms of protein production, cytotoxicity, and immunogenicity.

Initially, all vectors were complexed with an unmodified mRNA across a range of vector:mRNA ratios and screened for physicochemical properties. Physicochemical analysis showed that all polymeric and lipid-based vectors were able to fully complex the mRNA and had suitable properties for cellular uptake in terms of size, charge, and encapsulation efficiency (Figures 1 and 2). The zeta potential and encapsulation efficiency were dependent on the vector type as well as the vector:mRNA ratio used with polymeric materials showing an overall positive charge and full encapsulation and the lipid materials showing either a positive (e.g., jetMESSENGER) or negative charge (e.g., RNAiMax) with variable encapsulation efficiency. Although cationic materials are widely quoted as favorable for cellular transfection due to potential for electrostatic interaction with the anionic cell membrane, anionic nanoparticles have been successfully used for gene delivery particularly certain lipid-based materials.^39,46,47^ Therefore, vector systems could not be excluded based on the zeta potential alone. However, we were able to exclude certain formulations based on poor encapsulation of the mRNA (i.e., 1:1 mRNA:vector formulations for Lipofectamine reagents, RNAiMax and MessengerMAX). In terms of size, all particles were less than 200 nm, which has been previously quoted as favorable for cellular transfection.^40^

In terms of MSC transfection efficiency, we showed that overall, using a luciferase-expressing mRNA, lipid-based vectors provided higher levels of protein expression versus any of the polymeric materials that were tested in the study (Figure 4). To date, the delivery of mRNA has been dominated by lipid nanoparticles in all fields, so it is no surprise to see that in general, they give significantly higher expression compared to polymers in MSCs. Interestingly, no protein expression was detected with the highly branched polymers tested in this study, namely, branched PEI and Superfect (activated PAMAM dendrimer). We hypothesize that this may be in part due to the strong binding of mRNA to the highly branched materials, which is hindering the release of the nucleic acid intracellularly as indicated by the heparin dissociation studies (Figure 3). The concept of polymer complexes binding nucleic acid too tightly and preventing the nucleic acid cargo dissociation that is required for subsequent translation is not new and has been discussed before for both mRNA and pDNA nanoparticles.^42,48,49^ Similar to results observed here, Bettinger et al. failed to transfect a murine melanoma cell line (B16-F10 cells) with mRNA using polymer-based nanoparticles including Superfect and 25 kDa PEI but achieved successful translation using lipid-based systems.^49^ The group suggested that the lower electrostatic charge associated with lipid systems promotes dissociation and increases the translation efficiency. Furthermore, the group found that lower-molecular-weight polymers such as 2 kDa PEI permitted the release of mRNA and resulted in protein expression, although endosomolytic reagents were required to facilitate transfection. These findings suggest that higher-molecular-weight polymers that have been used previously with pDNA may hinder the release of mRNA. However, more systematic structural characterization studies would be needed to confirm this. Nevertheless, it is worth noting that some groups have achieved successful mRNA translation using larger polymeric vectors such as branched PEI, indicating that differing cell types and transfection protocols can also influence expression levels of different mRNA formulations.^23,24^

In this study, we directly compared the luciferase protein expression profile of three different mRNA types, unmodified mRNA, modified mRNA, and self-amplifying mRNA. Overall, we found that modRNA produced higher protein expression in MSCs versus uRNA or saRNA regardless of the dose or vector that is used. The improved efficiency of modRNA over uRNA has been well-established and is in line with previously published work.^11,12,50^ To the best of our knowledge, this is the first time that saRNA has been investigated for use in MSCs. Although protein expression was significantly lower versus the nonreplicating mRNA, some level of protein expression was achieved particularly with the polymeric vector jetPEI, indicating that it could have potential for certain applications. In the context of tissue engineering, achieving tailored and precise control over gene expression is of paramount importance. While screening for high expression levels is essential, it is equally crucial to consider the specific requirements of the tissue regeneration process. Therefore, it may be justifiable to bring forward nucleic acid cargos with differing/lower protein expression profiles to suit the unique demands of tissue engineering application. For example, in the context of bone regeneration, the delivery of multiple growth factors or genes encoding growth factors is a common approach to increase tissue regeneration.^51^ The pro-osteogenic BMP-2 is often codelivered with other growth factors such as the vascular endothelial growth factor (VEGF),^4,52^ fibroblast growth factor (FGF),^53^ platelet-derived growth factor (PDGF),^54^ and transforming growth factor (TGF-β1).^55^ Often, equal quantities of these growth factors are not necessary, and the use of saRNA could play a role where lower protein expression is required for certain growth factors. For instance, studies involving codelivery of recombinant BMP-2 and TGF-β1 have shown that TGF-β1 is delivered at 1/10 of the dose of BMP-2 for bone repair applications.^55,56^ However, further testing on saRNA and dose studies would be required to fully optimize its potential in tissue engineering.

Interestingly, the lipid-based RNA vectors showed poor mRNA translation when complexed with the saRNA molecule. We hypothesize that this may be due to the fact that these vectors are likely designed for smaller mRNA molecules (2000–5000 nt) unlike saRNA, which is around 9000 nt in length and again highlights the importance of optimizing vectors to specific mRNA cargos. A study by Blakney et al. highlighted something similar whereby the group found that vectors that work efficiently for nonreplicating mRNA cannot be directly translated for saRNA delivery.^57^ In the study, a range of poly(2-ethyl-2-oxazoline)/PEI copolymers were screened for pDNA, mRNA, and saRNA delivery to HEK 293T cells, and it was found that more highly charged, more hydrolyzed copolymers were favorable for saRNA delivery versus nonreplicating mRNA where a lower charge density was optimal.

The field of tissue engineering relies heavily on the use of biomaterial scaffolds to provide both mechanical and biological support for the growth of new tissues. These scaffolds typically serve to mimic the natural *in vivo* environment, allowing the infiltration and proliferation of cells to form new tissues. Another increasingly important feature of biomaterial scaffolds is their ability to support the loading and delivery of bioactive therapeutics such as growth factors or in this case nucleic acids such as mRNA. More and more, the field of tissue engineering is moving toward the use of gene therapies instead of recombinant proteins due to the high costs and unwanted side effects that have been associated with direct protein delivery.^58^ Gene-based scaffold approaches allow for a more controlled release of the bioactive molecule in a sustained yet transient manner.^59^ While pDNA-activated scaffolds have been traditionally used for growth factor delivery, mRNA-activated scaffolds have recently emerged as a potentially safer alternative, allowing transient growth factor expression without the risk of insertional mutagenesis. Despite the recent clinical success of mRNA-based therapeutics in the COVID-19 vaccines, its use in regenerative medicine remains limited. In this study, for a proof of concept, we chose to assess mRNA delivery from a collagen-nHA scaffold that has previously been loaded with pDNA and miRNA nonviral formulations and successfully applied for both *in vitro* and *in vivo* bone tissue engineering applications.^29,31,36,52^ Throughout this study, we demonstrated the ability of our collagen-nHA scaffolds to effectively deliver all three mRNA types to MSCs and achieve sustained protein expression over the course of at least 7 days. The level of expression appeared to be directly proportional to the amount of mRNA added to the scaffold with a 10 μg dose showing significantly higher RLUs compared to 2 and 5 μg doses for the first 3 days (Figure 5). Similar to this study, Wang et al. demonstrated the ability of a collagen-nHA scaffold to support the loading and release of a BMP-2/NS1 encoding uRNA for up to 10 days post soak-loading.^28^ Badieyan et al. also loaded modRNA encoding *Metridia* luciferase onto a commercially available collagen sponge and demonstrated sustained protein expression up to 11 days.^27^ However, unlike this study, a vacuum drying step of the collagen sponge was required in order to achieve the sustained release profile, which again highlights the advantage of our platform.

To further investigate the effects of the 3D environment on the mRNA expression profiles seen, Cy3-labeled jetPEI-modRNA (5 μg) nanoparticles were loaded onto the collagen-nHA scaffold and visualized using confocal microscopy. The results demonstrated that nanoparticles were evenly distributed across both surfaces of the scaffold where they had been initially soak-loaded. Within these nanoparticle-dense areas, nanoparticle distribution was widespread and homogeneous throughout the pores of the scaffold, which was important to elucidate given that the scaffold architecture has been specifically tailored to allow optimal infiltration and migration of cells throughout the pores.^60^ We hypothesize that this even distribution of nanoparticles near the surface would allow for the best possible contact with the cells regardless of where they were added or entered the scaffold. The fact that the mRNA nanoparticles initially adhered close to the surface where they were added could indicate an electrostatic interaction between the components of the scaffold and the mRNA nanoparticles. For example, the positively charged jetPEI nanoparticles are likely to interact with nHA nanoparticles, which have been reported previously by our group as anionic.^61^ We hypothesize that this electrostatic interaction could have beneficial effects in terms of nanoparticle retention and stability when translated into *in vivo* applications. In fact, Power et al. recently demonstrated using Cy3-tagged pDNA nanoparticles that our collagen-nHA scaffolds can retain nanoparticles for up to 28 days post soak-loading.^31^

Once we determined that our scaffold could support the delivery of mRNA to MSCs, we then sought to determine the optimal vector system for this application. We found that all four successful vectors (jetPEI, jetMESSENGER, RNAiMax, and MessengerMax) from the 2D monolayer transfection studies were also capable of delivering mRNA to MSCs when loaded onto the 3D collagen-nHA scaffold system (Figure 7). Overall, the lipid-based materials, in particular the two Lipofectamine reagents (RNAiMax and MessengerMax), showed the highest level of protein expression in the cells when combined with a base-modified mRNA on the scaffold. This is in line with previously published work as the majority of mRNA-activated collagen scaffolds for tissue engineering have utilized a lipid-based vector.^27,62−64^ Again, the saRNA nanoparticles led to low expression similar to what was observed in the 2D studies, indicating that it may not be the best suited mRNA type for our cell type or our application.

In order to confirm that the mRNA nanoparticles were not having any cytotoxic effects in MSCs within the scaffold, the LDH content and cell proliferation was assessed in modRNA scaffolds. LDH is an intracellular enzyme that is released upon damage to the cell membrane. Therefore, increased levels in cell culture media can indicate cytotoxic effects. Only jetPEI and jetMESSENGER-modRNA nanoparticles showed increased LDH at 24 h versus untreated cells, which again further validates the Lipofectamine reagents as an optimal system for *in vitro* screening of mRNA in MSCs. Notably, no cytotoxic effects were observed when jetMESSENGER-modRNA nanoparticles were used in the 2D studies. A possible explanation for this may be due to the increased contact time with cells within the scaffold. In 2D studies, the mRNA nanoparticles were removed after 4 h. However, in the scaffold studies, the nanoparticles remained on the scaffold throughout the study duration. Previous studies have indicated that contact time can have a direct impact on cell viability.^65^ In terms of the DNA content, we found no significant difference in any of the modRNA-loaded scaffolds versus unloaded scaffolds, indicating that the nanoparticles were not inhibiting cell proliferation within the scaffold.

Finally, the immunogenicity of the mRNA-activated scaffolds was assessed to determine the likelihood of inducing an inflammatory response if translated into *in vivo* studies (see the Supporting Information). In general, the PBMCs exposed to the MessengerMax-modRNA-loaded scaffolds expressed lower levels of IL-6, IL-8, and TNF-α secretion compared to the PBMCs exposed to the jetPEI-modRNA scaffolds, although this was only significant in the TNF-α scaffold at a 2 μg dose. Interestingly, increasing the dose of modRNA-vector nanoparticles did not significantly increase the cytokine response of the PBMCs in any of the groups. Similar results have been reported by Zhang et al., who demonstrated no significant differences in low (1.25 μg)- and high (5 μg)-dose modRNA matrices in terms of IL-1B and IL-6 secretion.^63^ However, the authors found dose-dependent differences in TNF-α and INF-y although this was not significant and varied depending on the time of analysis. It is difficult to directly compare studies on mRNA immunogenicity, as many will have different mRNA chemical modifications and differing vectors. The modifications on the mRNA used in this study (N1-methyl-pseudouridine substitution) have been well-established and indeed represent the gold standard of chemically modified mRNA having been utilized in both COVID-19 mRNA vaccines.^13,14^ Nevertheless, although no exogenously delivered mRNA can be considered immunologically inert, our findings highlight the significance of vector selection in determining the resulting immune response, and it should be carefully considered for future translational studies.

One potential limitation of this study is that only one type of collagen-based scaffold system was assessed for this initial proof-of-concept scaffold-mediated mRNA delivery. It is worth noting that many different variations of scaffolds are used, depending on the desired application and tissue type. It has been well-documented that varying scaffold compositions can alter protein expression kinetics in the case of DNA-loaded scaffolds.^30,37^ Therefore, it would be reasonable to assume that similar observations would be expected in mRNA-loaded scaffolds. For example, within our own group, Walsh et al. showed that luciferase expression can be observed for up to 28 days in a pDNA collagen-nHA scaffold versus only 14 days in a collagen-hydroxyapatite (HA) scaffold. The differences in expression are hypothesized to be linked to increased hydrogen bonding between the vector system (Superfect) and the higher concentration of HA in the collagen-HA scaffolds versus the collagen-nHA scaffolds. Therefore, the optimized conditions detailed in this study might require further optimization for translation to other scaffold types and further specific indication beyond bone. Nevertheless, the study still proves that with optimized transfection conditions, scaffold-mediated mRNA delivery has huge potential for tissue engineering applications.

In conclusion, we have thoroughly investigated the nonviral delivery of mRNA to MSCs for tissue engineering applications both in a 2D monolayer and from a collagen-based scaffold. After screening six different commercially available nonviral vectors and three mRNA types, we have determined that modified mRNA combined with lipid-based materials is optimal for these applications when high levels of protein expression are desired. It is hoped that this optimized mRNA-activated scaffold platform will serve as a versatile tool for researchers, offering a starting point to explore a range of clinically relevant therapeutic mRNAs in the field of tissue engineering. By using commercially available vectors in this study, our goal was to facilitate widespread adoption of this platform among researchers across different laboratories working on various tissue types. We anticipate that therapeutically relevant mRNAs can be easily incorporated into this optimized platform, which will allow researchers to further explore the enormous potential of mRNA in regenerative medicine.

## Supporting Information Available

Additional polydispersity index data for mRNA nanoparticle formulations investigated in this study (Table S1), MSC transfection data to determine optimal vector:mRNA ratios for MSC transfection (Figure S1), and supporting immunogenicity data in human peripheral blood mononuclear cells with leading mRNA nanoparticle formulations (Figure S2) (PDF)
