# Supplementary material for: Optimizing the Delivery of mRNA to Mesenchymal Stem Cells for Tissue Engineering Applications
Source: Mol Pharm. 2024 Mar 20;21(4):1662–76. doi: 10.1021/acs.molpharmaceut.3c00898 (PMC10988554; doi:10.1021/acs.molpharmaceut.3c00898)
Supplement: Supplementary file 1 — mp3c00898_si_001.pdf [file mp3c00898_si_001.pdf]

Optimising the delivery of messenger RNA to mesenchymal stem cells for tissue engineering applications.

Katie McCormick[1-3], Jorge Moreno Herrero[4], Heinrich Haas[4#], Sarinj Fattah[1,2], Andreas Heise[3,5,6], Fergal J. O'Brien[1,3,6,7], Sally-Ann Cryan*[1-3,6,7]

[1]Tissue Engineering Research Group, Department of Anatomy and Regenerative Medicine, RCSI, Dublin, D02 YN77, Ireland.
[2]School of Pharmacy and Biomolecular Sciences, RCSI, Dublin, D02 YN77, Ireland.
[3]Science Foundation Ireland Advance Materials and Bioengineering Research Centre, Dublin, D02 W9K7, Ireland.
[4], BioNTech SE, 55131 Mainz, Germany.
[5]Dept. of Chemistry, RCSI, Dublin, D02 YN77, Ireland.
[6]Science Foundation Ireland Centre for Research in Medical Devices, Galway, H91 W2TY, Ireland.
[7]Trinity Centre for Biomedical Engineering, Trinity College Dublin, Dublin, D02 R590, Ireland.
[#] Current address: Department of Biopharmaceutics and Pharmaceutical Technology, Johannes Gutenberg-University, 55128 Mainz, Germany.

Supporting Information

Table S1: Polydispersity index (PDI) of mRNA nanoparticle formulations. Each of the six different non-viral vectors (Branched PEI, Superfect®, jetPEI®, jetMESSENGER®, RNAiMAX® and MessengerMax®) was complexed with 1µg uRNA across three different N/P or mass ratios in nuclease-free water and PDI was measured using a Zetasizer.

Non-viral vector	Vector:mRNA ratio (v/w) or N/P ratio	Polydispersity Index (PDI)
**25kDA Branched PEI**	N/P 5	0.23
	N/P 7	0.26
	N/P 10	0.27
**Superfect®**	2:1	0.20
	5:1	0.27
	10:1	0.35
**jetPEI®**	N/P 5	0.28
	N/P 7.5	0.38
	N/P 10	0.33
**jetMESSENGER®**	1.6:1	0.35
	2:1	0.36
	2.4:1	0.33
**RNAiMax®**	1:1	0.35
	1.5:1	0.30
	3:1	0.33
**MessengerMax®**	1:1	0.38
	1.5:1	0.54
	3:1	0.36

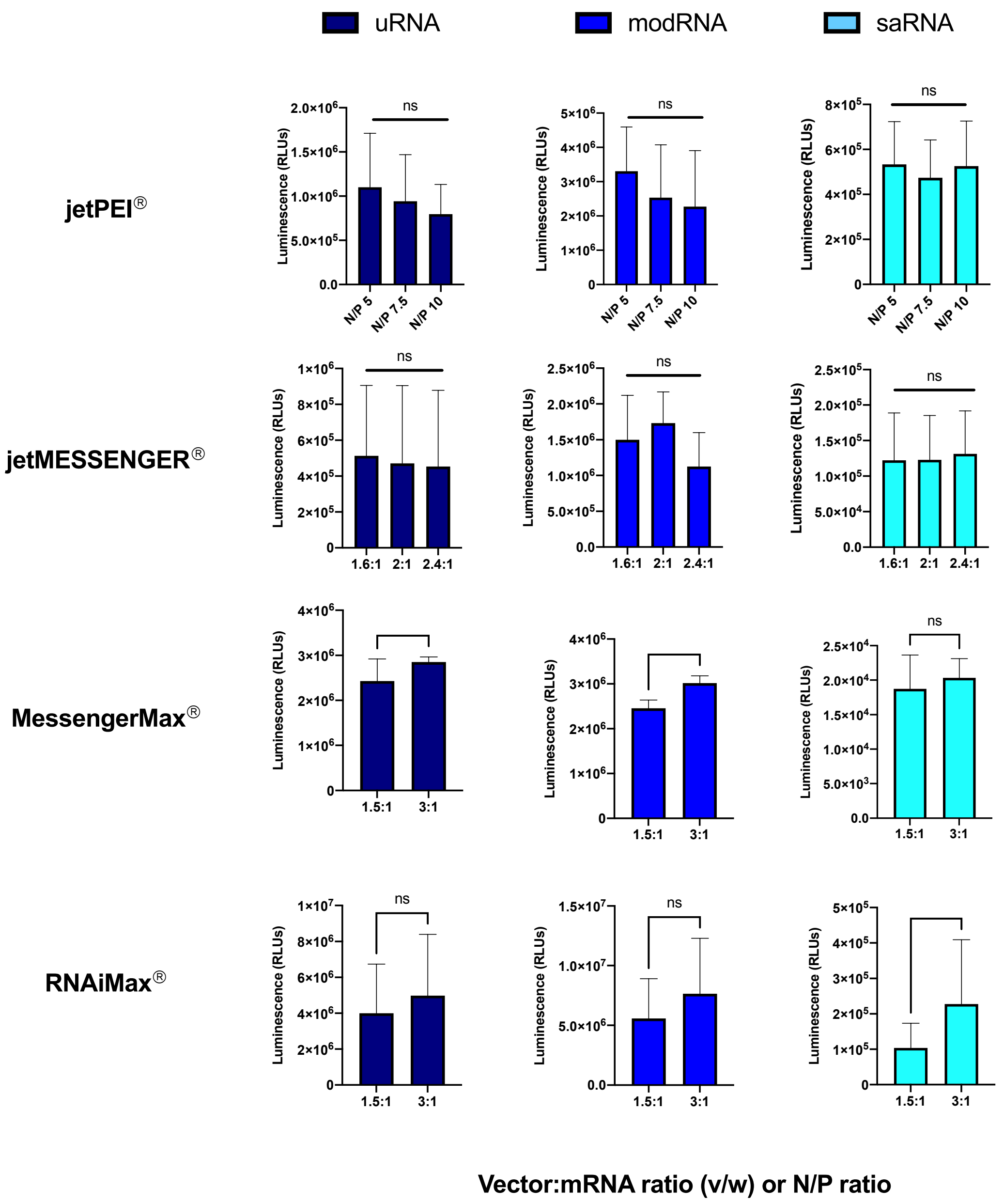

Figure S1. Optimisation of vector:mRNA ratio for MSC transfection. Four different non-viral vectors (jetPEI®, jetMESSENGER®, MessengerMax® and RNAiMAX®) were complexed with each of the three firefly luciferase mRNA types – unmodified mRNA (uRNA), base-modified mRNA (modRNA) and self-amplifying mRNA (saRNA) – across a range of vector:mRNA (v/w) or nitrogen/phosphate (N/P) ratios and used to transfect MSCs. Luciferase expression was analysed 24 hours post-transfection. The following ratios were deemed optimal for each vector; N/P 5 for jetPEI-mRNA, 2:1 v/w for jetMESSENGER-mRNA and 3:1 v/w for MessengerMax® and RNAiMAX-mRNA nanoparticles. Results are expressed as mean ± SD (n=3) where *p<0.05, ****p<0.0001, ns = not significant.

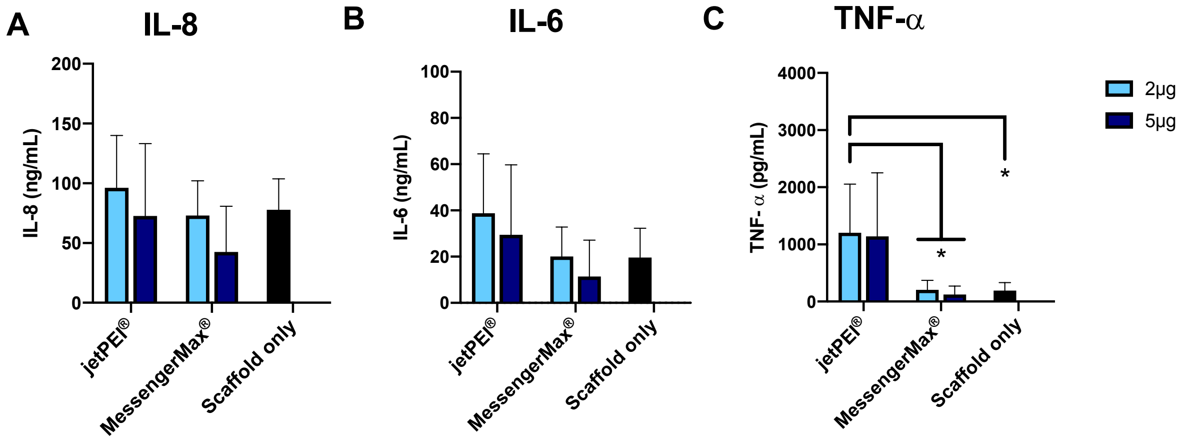

Figure S2. Cytokine expression in hPBMCs post-exposure to modRNA activated scaffolds. modRNA encoding firefly luciferase was loaded onto collagen-nHA scaffolds at two different doses (2µg and 5µg) complexed with either jetPEI® (N/P 5) or MessengerMax® (3:1 v/w) and (A) IL-8 (B) IL-6 and (C) TNF- α content was evaluated at 24 hours. Results are expressed as mean ± SD (n=3) where * $p<0.05$.
